# Whole-Genome-Based Public Health Surveillance of Less Common Shiga Toxin-Producing Escherichia coli Serovars and Untypeable Strains Identifies Four Novel O Genotypes

**DOI:** 10.1128/JCM.00768-19

**Published:** 2019-09-24

**Authors:** Christina Lang, Miriam Hiller, Regina Konrad, Angelika Fruth, Antje Flieger

**Affiliations:** a Division of Enteropathogenic Bacteria and Legionella, National Reference Centre for Salmonella and Other Enteric Bacterial Pathogens, Robert Koch Institute, Wernigerode, Germany; b Department of Infectiology, Bavarian Health and Food Safety Authority, Oberschleissheim, Germany; Medical College of Wisconsin

**Keywords:** genoserotyping, public health surveillance, STEC, whole-genome sequencing

## Abstract

Shiga toxin-producing Escherichia coli (STEC) and the STEC subgroup enterohemorrhagic E. coli cause intestinal infections with symptoms ranging from watery diarrhea to hemolytic-uremic syndrome (HUS). A key tool for the epidemiological differentiation of STEC is serotyping. The serotype in combination with the main virulence determinants gives important insight into the virulence potential of a strain. However, a large fraction of STEC strains found in human disease, including strains causing HUS, belongs to less frequently detected STEC serovars or their O/H antigens are unknown or even untypeable. Recent implementation of whole-genome sequence (WGS) analysis, in principle, allows the deduction of serovar and virulence gene information. Therefore, here we compared classical serovar and PCR-based virulence marker detection with WGS-based methods for 232 STEC strains, focusing on less frequently detected STEC serovars and nontypeable strains. We found that the results of WGS-based extraction showed a very high degree of overlap with those of the more classical methods. Specifically, the rate of concordance was 97% for O antigens (OAGs) and 99% for H antigens (HAGs) of typeable strains and >99% for *stx*_1_, *stx*_2_, or *eaeA* for all strains. Ninety-eight percent of nontypeable OAGs and 100% of nontypeable HAGs were defined by WGS analysis. In addition, the novel methods enabled a more complete analysis of strains causing severe clinical symptoms and the description of four novel STEC OAG loci. In conclusion, WGS is a promising tool for gaining serovar and virulence gene information, especially from a public health perspective.

## Abstract (precis)

Shiga toxin-producing Escherichia coli (STEC) and the STEC subgroup enterohemorrhagic E. coli cause intestinal infections with symptoms ranging from watery diarrhea to hemolytic-uremic syndrome (HUS). A key tool for the epidemiological differentiation of STEC is serotyping. The serotype in combination with the main virulence determinants gives important insight into the virulence potential of a strain.

## INTRODUCTION

Shiga toxin-producing Escherichia coli (STEC) strains, including the STEC subgroup enterohemorrhagic E. coli (EHEC), cause intestinal infections ranging from sporadic disease to large outbreaks worldwide (1). In Germany, about 2,000 cases of STEC-associated diarrhea/bloody diarrhea and about 70 cases of severe hemolytic-uremic syndrome (HUS) have been reported annually since 2015. Of note, the number has been steadily increasing in recent years, a tendency which is observed throughout Europe (2, 3).

The most important virulence determinant of STEC/EHEC strains is Shiga toxin (Stx). Stx is responsible for severe pathologies like HUS and is divided into two different types (4). Stx1 has three subtypes, namely a, c, and d, whereas the more toxic Stx2 is represented by eight different subtypes, designated a to h. Subtyped *stx* genes are important epidemiological markers. Additionally, disease outcome has been attributed to specific Stx types. HUS-associated strains (e.g., strains from the HUS-associated enterohemorrhagic E. coli collection [HUSEC]) often carry the *stx*_2a_, *stx*_2d_, *stx*_2c_, and *stx*_1a_ genes alone or in combination with other types (5, 6). *stx* gene subtyping and detection of other virulence determinants may therefore permit risk profiling of such pathogens (5). Further virulence factors/genes are present in so-called classical STEC strains but are absent in a variety of other often less characterized STEC strains. Examples are a type III protein secretion system coded on a pathogenicity island, namely, the locus of enterocyte effacement (LEE), and the enterohemolysin HlyA, encoded by the gene *ehxA*. LEE induces intimate attachment of the bacteria to the intestinal epithelia, and HlyA is a pore-forming toxin (4, 7).

A key tool for the differentiation of STEC is serotyping. Classical STEC serotyping has routinely been performed for more than 50 years, and assignment of a serovar is important for surveillance and cluster detection. The O and H surface antigens, specifically, the lipopolysaccharide (LPS) and flagellin of the bacteria, respectively, are typically used for subdifferentiation (8). So far, 182 O serogroups (O1 to O188, except for O31, O47, O67, O72, O93, and O94) and 53 associated H forms (H1 to 56, except for H13, H22, and H50) have been described (9). Interestingly, only strains of a few O antigen (OAG) types, such as O91, O103, O146, O157, O26, O113, O128, O76, and O145, often in combination with specific H antigens (HAGs), cause more than 50% of STEC infections (1, 9). Of these more frequently found serogroups, O157 is principally associated with the development of severe disease (1, 10).

However, it is important to note that a large fraction (about 30%) of HUSEC strains does not consist of frequently found STEC OAG types (6). In addition, the 2011 HUS outbreak in Germany, caused by an STEC isolate of the rare serovar O104:H4, illustrates the strong potential of these more unusual strains to cause severe disease. It was the largest outbreak of bloody diarrhea/HUS detected so far worldwide and involved 53 deaths, 833 HUS cases, and about 3,000 cases of gastroenteritis (11–13).

Implementation of whole-genome sequencing (WGS) techniques into public health microbiology now permits genome-based typing for pathogen surveillance and cluster analysis. The new method also enables deduction of serovar information (14–19). This is especially important for previously nonserotypeable strains, namely, for rough, nonmotile (nm), and O nontypeable (Ont)/H nontypeable (Hnt) strains. Joensen et al. (16) created a FASTA database of specific O antigen processing systems and flagellin genes for O and H typing, respectively. This resource is a component of the publicly available web tool hosted by the Center for Genomic Epidemiology (CGE; DTU, Denmark; http://www.genomicepidemiology.org). They analyzed data for ∼500 to 600 E. coli whole-genome sequences with serotype information with the SerotypeFinder CGE tool. The O and H types were consistently predicted by classical serotyping in 560 of 569 cases and 504 of 508 cases, respectively. The authors therefore concluded that E. coli serotyping can be done solely from WGS data and that WGS provides a superior alternative to conventional serotyping (16). Further, Chattaway et al. (19) evaluated the use of WGS for routine public health surveillance of non-O157 STEC isolates by comparing this approach to phenotypic serotyping. Of the 102 isolates, concordant results between methods were found for 98. The most common non-O157 STEC serogroups detected were O146 and O26. Thirty-eight isolates could not be phenotypically serotyped. Only one of these was not successfully serotyped using the WGS data (19).

In the study presented here, we compared classical serovar analysis with WGS-based genoserotyping in a setting for the routine analysis of STEC isolates of the German National Reference Centre for *Salmonella* and Other Enteric Bacterial Pathogens (NRC). Whereas previous studies mostly concentrated on strains with more common OAGs, we focused on less frequently detected STEC serovars and nontypeable strains. In addition, we compared PCR-based virulence gene analysis with WGS-based data. As a conclusion, we found a very high degree of overlap of the results of WGS with the results of classical or PCR-based methods. In addition, the novel methods enabled further analysis of strains causing severe clinical symptoms and the description of four novel STEC OAG loci.

## MATERIALS AND METHODS

### Strains.

The strains used in the study are listed in Table S1 in the supplemental material. All strains were human isolates, except for seven food isolates. Strains were grown on nutrient agar (Oxoid GmbH, Germany) or in tryptic soy broth (TSB; BD-BBL, Germany), if not stated otherwise. Testing for enterohemolysin production was performed on enterohemolysin agar (Sifin GmbH, Germany).

### E. coli serotyping.

Serotyping was performed using antisera against E. coli O antigens 1 to 188 and E. coli H antigens 1 to 56 by use of a microtiter agglutination method, as described elsewhere (20).

### Antibiotic susceptibility testing.

All of the strains were tested for susceptibility to 16 antibiotics according to EUCAST recommendations for E. coli by a broth microdilution assay (http://www.eucast.org/fileadmin/src/media/PDFs/EUCAST_files/Disk_test_documents/2019_manuals/Reading_guide_BMD_v_1.0_2019.pdf).

### PCR-based virulence gene analysis.

All *stx* genotypes and the presence of *eae* (encoding adhesin intimin) and the *ehxA* gene were first determined using PCR (5, 21).

### WGS.

Whole-genome sequencing (WGS) was accomplished using short-read paired-end sequencing with the MiSeq (2 × 300 bp) and HiSeq 1500 (2 × 250 bp) instruments (Illumina, San Diego, CA). For this, DNA from the E. coli strains was isolated with a Qiagen DNeasy blood and tissue kit (Qiagen) according to the manufacturer’s instructions, and 1 ng of the extracted DNA was used to generate libraries by using the Nextera XT DNA library according to the manufacturer’s instructions (Illumina, San Diego, CA). Requirements for the sequence raw data were as follows: sequence yield, >600,000 reads/sample; mean sequence quality score (Phred score), >25; and genome coverage, >30-fold. On average, the sequence yield was about 2.6 million reads/sample, and the genome coverage was 120-fold.

### Bioinformatics analyses.

Raw reads were subjected to quality control and trimming by use of the QCumber pipeline (version 2.1.1; https://gitlab.com/RKIBioinformaticsPipelines/QCumber) utilizing the FastQC (version 0.11.5; https://www.bioinformatics.babraham.ac.uk/projects/fastqc/), Trimmomatic (version 0.36 [22]), and Kraken (version 0.10.6 [23]) tools. Trimmomatic was used with the default parameters (Phred score, 33). On average, 80% of the reads remained after trimming. To identify the serotype and virulence genes, the trimmed reads were mapped by means of the standard Geneious assembler (settings, medium sensitivity and no iterations; Geneious, version R10.0.5; Biomatters Ltd.) against the respective reference sequence. Requirements for positive matches were 100% coverage of the reference sequence, >90% identity with the reference sequence, and high quality for >90% bases in the sequence.

Reference sequences for the *wzm*, *wzy*, *wzm*, *wzt*, and *fliC* genes for serotype determination and for virulence marker genes were downloaded from the Center for Genomic Epidemiology (CGE; DTU, Denmark; SerotypeFinder, VirulenceFinder; https://cge.cbs.dtu.dk/services/data.php). Further reference sequences for serotyping were obtained from NCBI (Table S2).

Ridom SeqSphere+ software (version 5.1.0; Ridom GmbH, Germany) was used to create a neighbor-joining tree, based on 2,513 targets from the E. coli core-genome multilocus sequence typing (cgMLST) EnteroBase, by pairwise analysis, ignoring missing values. Ridom SeqSphere+ software was also used to determine multilocus sequence typing (MLST) Warwick sequence types (STs).

When potentially novel O antigen loci were analyzed, the reads were *de novo* assembled with the program A5 (version 2.1.3), and the contigs were further analyzed by means of Geneious software. Using all known O antigen clusters (OAGCs) as the annotation reference (Geneious tool, annotate from), the partially annotated contigs were extracted and the OAGC region was defined to be between the genes *galF* (encoding UTP-glucose-1-phosphate uridyltransferase) and *hisI* (encoding the histidine biosynthesis bifunctional protein), because genes required for the biosynthesis of E. coli OAGs are mostly located at this site (24–27). Some open reading frames (ORFs) of the new clusters could not be annotated using the known OAGCs as a reference. These were then translated into proteins, and analysis with the NCBI pBLAST program (standard settings; database, nonredundant protein sequences; algorithm, blastp) was performed to search for functional homologues. Homologues of the newly defined OAGCs were detected using the NCBI nBLAST program (standard settings; database, nucleotide collection; optimized for highly similar sequences).

### Data availability.

The annotated sequences of the new OGACs were uploaded to NCBI (GenBank accession numbers MN172354 to MN172357). The raw FASTQ sequences were uploaded to the European Nucleotide Archive (ENA) under study accession number PRJEB32361.

## RESULTS

### STEC isolates analyzed at NRC from 2015 to 2017 and selection of strains for WGS, focusing on less frequently found serovars and untypeable O antigens.

**FIG 1:**
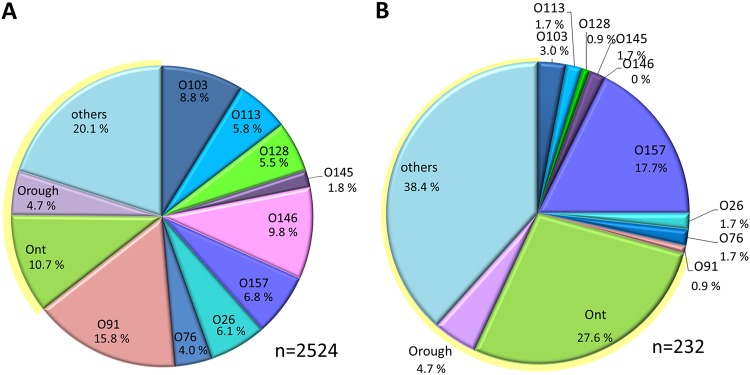
Classically determined serotypes of all NRC STEC strains (A) and of the 232 strains chosen for WGS (B). The strains were recovered from 2015 to 2017.

NRC receives STEC samples from human disease cases for further subtyping. In 2015, 2016, and 2017, ∼641, ∼895, and ∼1,466 STEC samples (total, 3,002), respectively, were obtained. These represent samples from about 40 to 74% of the reported STEC infections per year (2, 9). Of these, about 84% were serotyped by the classical microtiter agglutination method, and all were analyzed by PCR for the presence of virulence genes, such as *stx*_1_, *stx*_2_, and *eaeA*. Sixty-three percent of the STEC isolates obtained from 2015 to 2017 belonged to the most frequently identified OAG types, including O26, O91, O76, O103, O113, O128, O145, O146, and O157. Approximately 15% were Ont or had a rough LPS (Orough) type, and a further ∼20% did not belong to the above-mentioned frequently found serovars (Fig. 1A). All isolates harbored *stx*, and of these, 41.5% had *stx*_1_, 34.1% had *stx*_2_, and 24.4% had both *stx*_1_ and *stx*_2_. Of the STEC isolates obtained from 2015 to 2017, 30.5% possessed the *eaeA* gene; specifically, 49.0% possessed *eaeA* in combination with *stx*_1_, 37.4% possessed *eaeA* in combination with *stx*_2_, and 13.4% possessed *eaeA* in combination with both *stx*_1_ and *stx*_2_.

Next, we selected 232 STEC strains among isolates recovered from 2015 to 2017 by use of the following criteria: (i) they had less frequently found (less common) OAGs, (ii) they had uncommon O:H combinations, and (iii) they were Ont/Orough types. For comparison, about 25% of the strains that we included had more common OAGs. The serovar distribution is shown in Fig. 1B. Additionally, antibiotic susceptibility testing was performed for epidemiological purposes, and analysis revealed that 70% of the selected strains were susceptible and 21% were resistant to more than one of the tested antibiotics (see Table S1 in the supplemental material).

### The O antigens determined by WGS highly correlate with those determined by classical serotyping.

OAG types were extracted from the genome sequence data for the 232 selected strains. Here, we mapped the trimmed reads against a set of reference sequences (see Materials and Methods). By means of classical serotyping, in 67.2% of the strains the OAG was typeable, and among these strains, the OAG type of 96.8% was confirmed by WGS analysis. Only five strains (3.2%) showed a discordant result.

Specifically, two strains (16-01717 and 16-01865) were classically serotyped as O57, but WGS analysis for the first one yielded OgN1, which was recently assigned as a new OAG type (18), and WGS analysis for the second one yielded O2. One strain (16-04148) was determined to be O169 but was typed as O81 by WGS. The fourth strain (17-05507) was originally serotyped as O109, but WGS analysis revealed that it was O182. The sequence of the last strain (16-04178), defined as O54, did not sufficiently match any reference sequence and might belong to a novel OAG cluster (see below).

For 76 (32.8%) of the 232 strains, OAG was not typeable (28.0%) by classical serotyping or was Orough (4.7%). The majority (97.8%) could be classified by WGS analysis, and the most frequently found types were O27 (9 strains), O100 (7 strains), O80 (4 strains), and O153/178 (4 strains) (Table S1). About 15% of the nontypeable strains belonged to the recently described OgN O antigen clusters (OAGCs; OgN1, OgN10, OgN12, OgN13, OgN31) (18). Most interestingly, the sequences of five strains did not match known OAG loci, and therefore, these strains might belong to novel OAGs (see below). Two of the strains harbored a similar OAG locus. In summary, the serotype data extracted from WGS correlate very well with the serotype data obtained by classical methods, and WGS allows classification of so far untypeable strains and the identification of novel O genotypes.

### The H antigens determined by WGS highly correlate with those determined by classical serotyping.

Next, we extracted the H types from the WGS data. By classical serotyping, the HAG was typeable in 80% of the strains, and in 99% of these strains, the type was confirmed by WGS analysis. Only two strains showed noncorrelating results (strain 16-01506, which was H14 by WGS and H19 by classical serotyping, and 17-05292, which was H31 by WGS and H36 by classical serotyping). By means of classical serotyping, 2.6% HAGs were untypeable and 17.9% of the strains were not motile (total, 20.5%). All of these were defined by WGS analysis.

### Identification of four novel O antigen gene loci.

**FIG 2:**
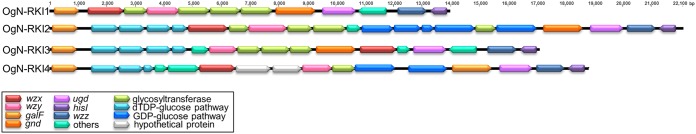
Four novel O antigen gene clusters (OgN-RKI1 to OgN-RKI4) identified in this study.

As mentioned above, the OAGCs of 6 strains were not identified because they did not match any known OAG loci (Table 1). As they might be new OAG loci, *de novo* assembly of the MiSeq reads was performed, and the resulting contigs were annotated using all known OAGC sequences as a reference (see Materials and Methods for details). Some ORFs of the new clusters could not be annotated using the known OAGCs as a reference. These were then translated into proteins, and pBLAST analysis was performed to search for functional homologues. By this means, it was possible to define new putative O-unit-processing genes, specifically, *wzx* (the gene for O antigen flippase) and *wzy* (the gene for O antigen polymerase), which are relatively unique for each individual O type (28). Indeed, four novel OAGCs were defined; two strains carried identical OgN-RKI1 OAGCs (strains 16-01174 and 16-04846), another two strains carried identical OgN-RKI2 OAGCs (strains 16-02258 and 17-05936), and the remaining two strains were assigned to OgN-RKI3 (strain 16-04178) and OgN-RKI4 (strain 17-05676) (Table 1). Figure 2 gives an overview of these new OAGCs.

**TABLE 1:** Serotype, MLST ST, and virulence gene profile of the six STEC strains with novel OAGCs

RKI no.	OAG type	HAG type	MLST ST	Presence of the following gene:													
				*stx*_1_^*a*^	*stx*_2_^*a*^	*eaeA*	*hlyA*	*espP*	*fyuA*	*iha*	*irp2*	*catP*	*lpf_O26_*	*lpf_O113_*	*subAB*	*terA*	*EAST1*
16-01174	OgN-RKI1	49	9300	−	+/b	−	+	−	−	+	−	−	−	−	+	+	+
16-04846	OgN-RKI1	20	6060	+/c	−	−	−	−	−	−	−	−	−	−	−	−	−
16-02258	OgN-RKI2	16	336	+/c	−	−	−	−	−	−	−	+	+	−	−	−	−
16-04178	OgN-RKI3	21	155	−	+/a	−	−	−	−	−	−	−	−	+	−	−	−
17-05676	OgN-RKI4	29	515	−	+/b	−	−	−	−	+	−	−	−	−	−	−	+
17-05936	OgN-RKI2	16	336	+/c	−	−	−	−	−	−	−	+	−	−	−	−	+

a The letters after the slashes indicate the *stx*_1_ or *stx*_2_ subtype.

**FIG 3:**
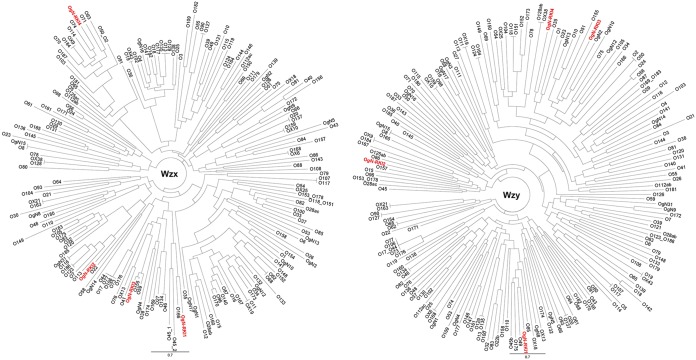
Phylogenetic analysis of Wzx and Wzy homologs of the four novel OAGCs (OgN-RKI1 to OgN-RKI4) (red) and E. coli O serotype, OX group, and OgN group reference strains based on amino acid sequences.

Comparison of the Wzx and Wzy protein sequences to those from 167 O serogroup strains, 10 OX group reference strains, and 15 OgN strains indicated that the sequences of the new OAGCs were unique compared to the sequences of known OAGCs (29) (Fig. 3).

### Phylogenetic analysis revealed that many isolates clustered according to their serotypes.

**FIG 4:**
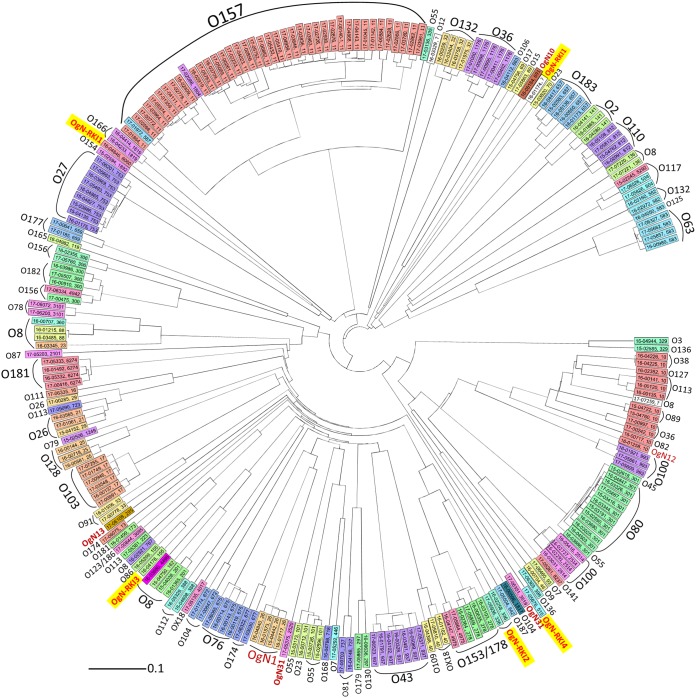
Chromosomal phylogeny of 232 STEC strains whose genomes were sequenced represented as a neighbor-joining tree and its relation to the serogroup and 7 gene MLST types. Ridom SeqSphere+ was used to create the neighbor-joining tree, based on 2,513 targets from the E. coli cgMLST EnteroBase, by pairwise analysis, ignoring missing values. Labels containing the strain number and the MLST type separated by a comma. Different colors are assigned to distinct MLST types. OAGs are depicted in the outer circle. The new OAGs found in this study are highlighted in yellow with red text. Further new OAGs (OgN) found by WGS are also labeled in red.

We further analyzed whether there was a correlation between the O antigen extracted from WGS and the assigned MLST type or/and the chromosomal phylogeny determined by means of cgMLST (Fig. 4). For most of the strains showing the same serotype (3 to 10 strains), the MLST type correlated with the O antigen type throughout, for example, for O103:H2 (sequence type 17 [ST17]), O128:H2 (ST25), and OgN1 (ST26 (Fig. 4). The O157:H7 strains belonged to ST11 in a major way; however, in two cases, the MLST types were ST587 and ST1804. Figure 4 shows that these two O157:H7 strains were located in the same phylogenetic branch with the other ST11 strains, and therefore, all of the strains were closely related. This was also the case for several other serotypes belonging to different MLST types, like O26:H11 (ST21, ST29), O153/178:H7 (ST278, ST4975), O117:H7 (ST504, ST5292), and O156:H25 (ST300, ST4942) (Fig. 4).

Conversely, strains sharing the same OAG but harboring different HAGs belonged to different MLST types and distinct phylogenetic branches, like O18:H2/21 (ST4017, ST40), O36:H19/14 (ST10, ST1176), and O55:H7/9/12 (ST335, ST301, ST101). It is interesting that the branch of strains belonging to MLST ST10 comprised a large diversity of serotypes, including O89:H9, O113:H4, O82:H4, OgN12:H32, O127:H40, O38:H26, and O36:H19 (Fig. 4). On the other hand, O8 antigen strains belonged to a variety of different MLST types and occurred at different branches in the phylogenetic tree (ST23, ST88, ST136, ST162, ST767, ST201, and ST4496), and several O8 strains with the same HAG did not even belong to the same MLST type (Fig. 4). The four novel O genotypes identified here were found at different branches, whereby the two strains sharing OgN-RKI2 were closely related; however, the two strains sharing OgN-RKI1 were not. To summarize, the MLST type gives insight into the phylogenetic relationship of a large fraction of STEC strains; however, depending on the serotype, it does not completely reflect the serotype or genomic phylogeny.

### WGS-based virulence gene determination highly correlates with PCR-based data.

**FIG 5:**
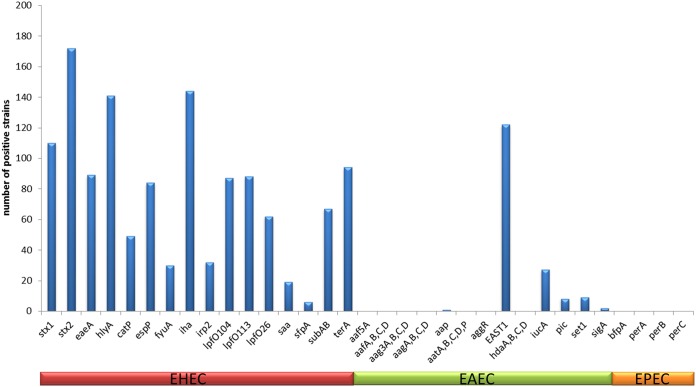
Summary of selected E. coli pathovar virulence genes extracted from the 232 STEC strains and analyzed by WGS.

From the genome sequences, we further extracted 27 EHEC, enteropathogenic E. coli (EPEC), and enteroaggregative E. coli (EAEC) virulence gene markers and 6 gene loci (loci for the EAEC AAF-I to AAF-IV genes, the *aat* operon, and the *ehx* operon). We observed 99 to 100% concordance with PCR-derived data concerning STEC markers *stx*_1_, *stx*_2_, *eaeA*, and *hlyA*, confirming the high level of suitability of the PCR-based methods. The presence of the *stx*_1_ gene in one strain was, however, not confirmed by the WGS data. This might be due to the loss of the *stx*_1_ phage in this strain. Further, the *stx*_2_ genes in two strains and the *ehxA* genes in four strains were found by WGS analysis but were missed by the PCR method. Figure 5 shows the distribution of selected STEC/EHEC, EPEC, and EAEC virulence gene markers detected by WGS analysis. Interestingly, the heat-stable enterotoxin 1 (EAST1) gene was present in more than 50% of the STEC strains analyzed here.

### Strains causing HUS.

Among the strains analyzed by WGS, 14 were isolated from cases with HUS or fatal cases (Table 2). Nine of those belonged to more frequently found STEC OAGs, such as O26:H11 (two strains), O103:H2 (two strains), O113:H21, O145:H28 (two strains), O157:H7, and O157:Hnm; all of these are present in HUSEC (6). A further five strains belonged to less frequently found STEC OAGs. The serotypes were O55:H7, O80:H2, O174:H21, and O177:H25 (two strains). Except for the O80 and O177 strains, all serovars are present in HUSEC (6). Eight of the 14 strains harbored *stx*_2a_, 3 strains harbored *stx*_2c_, 2 strain harbored *stx*_2d_, and 2 strains harbored *stx*_1a_. The last two strains, which were of serotype O103:H2, did not have an additional *stx*_2_ gene (Table 2).

**TABLE 2:** Serotype, MLST ST, and virulence gene profile of EHEC strains causing HUS or death

OAG type	HAG type	MLST ST	RKI^*a*^ strain no.	Clinical findings	Presence of the following gene:																					
					*stx*_1_^*b*^	*stx*_2_^*b*^	*eaeA*	*hlyA*	*espP*	*fyuA*	*iha*	*irp2*	*catP*	*lpf_O26_*	*lpf_O113_*	*sfpA*	*subAB*	*terA*	*EAST1*	*iucA*	*plc*	*set1*	*sigA*	*aap*	*aatA*	*aagA, aagC*
26	11	29	17-00285	HUS	−	+/a	+	+	+	+	+	+	−	+	+	−	−	+	−	−	−	−	−	−	−	−
26	11	21	17-01061	HUS	−	+/a	+	+	+	+	+	+	+	+	+	−	−	+	+	−	−	−	−	−	−	−
55	7	335	17-03136	HUS	−	+/a	+	−	−	−	−	−	−	−	−	−	−	−	+	−	−	−	−	−	−	−
80	2	301	16-03025	HUS	−	+a	+	+	+	−	+	−	−	−	−	−	−	+	−	−	−	−	−	−	−	−
103	2	17	17-01749	HUS	+/a	−	+	+	−	−	+	−	−	+	−	−	−	+	−	+	−	−	−	−	−	−
103	2	17	17-03548	HUS	+/a	−	+	+	−	−	+	−	−	+	−	−	−	+	−	+	−	−	−	−	−	−
113	21	223	17-05381	HUS	−	+/a	−	+	+	−	+	−	−	−	+	−	+	−	−	−	−	−	−	−	−	−
145	28	32	16-03404	HUS	−	+/a	+	+	+	−	+	−	−	−	−	−	−	+	+	−	−	−	−	−	−	−
157	7	11	17-01864	HUS	−	+/a	+	+	−	−	−	−	−	−	−	+	−	−	+	−	−	−	−	−	−	−
157	7	587	17-01972	HUS	−	+/a	+	+	−	−	−	−	−	−	−	+	−	−	+	−	−	−	−	−	−	−
174	21	677	17-03030	HUS	−	+/d	−	−	−	+	−	−	−	+	+	−	−	−	−	−	−	−	−	−	−	−
177	25	659	17-00641	HUS	−	+/c	+	+	+	−	+	−	+	−	+	−	−	+	+	+	−	−	−	−	−	−
177	25	659	17-01185	Death/HUS	−	+/c	+	+	+	−	+	−	+	−	+	−	−	+	+	+	−	−	−	−	−	−
145	28	32	17-01975	Death	−	+/c	+	+	+	−	+	−	−	−	−	−	−	+	+	−	−	−	−	−	−	−

a RKI, Robert Koch Institute.

b The letters after the slashes indicate the *stx*_1_ or *stx*_2_ subtype.

### Correlations of *stx* subtypes with O antigens.

We used the WGS data to get an overview about the *stx* gene subtypes carried by our study strains. For 109 *stx*_1_-positive strains, the *stx*_1a_ subtype was found in 55.9%, *stx*_1c_ was found in 42.2%, and *stx*_1d_ was found in 1.8%. The *stx*_2_ gene was detected in 174 strains, and the subtype distribution was as follows: 32.0% *stx*_2a_, 29.8% *stx*_2b_, 13.0% *stx*_2c_, 4.5% *stx*_2d_, 12.6% *stx*_2e_, 4.6% *stx*_2f_, and 2.9% *stx*_2g_. For example, all O103, O117, and O182 strains carried *stx*_1a_. *stx*_1c_ was found in all O38, O43, O78, O112, and O153/178 strains. *stx*_2a_ was detected in all O26, O145, and OgN31 strains; *stx*_2b_ was detected in all O2, O110, and OgN1 strains; *stx*_2e_ was detected in all O89 and O100 strains and the majority (9 of 12) of O8 strains; *stx*_2f_ was detected in all O63 and O132 strains; and *stx*_2g_ was detected in most (5 of 7) of the O36 strains. Among the O157:H7 strains, *stx*_1a_ alone was found in 2.4%, *stx*_2a_ alone was found in 34.1%, *stx*_2c_ alone was found in 19.5%, and the combination of *stx*_1a_ and *stx*_2a_ or *stx*_1a_ and *stx*_2c_ was found in 14.6% and 29.3%, respectively.

## DISCUSSION

In this study, we preferentially analyzed STEC strains showing (i) less frequently detected STEC OAGs, (ii) uncommon O:H combinations, and (iii) Ont/Orough types. As described above, 35% of isolates analyzed at NRC usually belong to these categories; in this study, we doubled this portion to 70% (Fig. 1A). We set out to compare the results of classical serotyping and PCR-based detection of main virulence markers with WGS-derived findings and put those into context with STEC isolates belonging to the more frequently found OAGs. Validation of WGS-based methods for the strains predominately selected here is especially important, since a huge variety of such strains exist, and so far, the vast majority of studies have studied only the more common STEC types. Uncommon types induce a substantial percentage of severe disease and large outbreaks and therefore deserve special attention (6, 9, 12, 13).

In the genomic era, OAG serotyping remains an important epidemiological marker of STEC used as a first indication of strain virulence (30). Therefore, it is important to serotype untypeable and rough strains, which is now possible by using genome analysis. Our study shows that WGS data can be used to extract STEC serotypes and virulence markers for the selected strains, yielding for about 97 to 99% of the strains results concordant with those of the more classical methods. Importantly, classification of nontypeable or rough strains was possible by WGS, and WGS even allowed the identification of novel OAG genotypes.

In this study, we identified four novel OAG genotypes of six strains, which were found to be located on five distinct phylogenetic branches (Fig. 4; Table 1). nBLAST analysis of the novel OAGCs revealed that OgN-RKI1 is abundant in Shigella boydii serovar 19 with a nucleotide identity of over 98% and in one published STEC strain with an untypeable OAG (see Table S3 in the supplemental material). This shows that OgN-RKI1 is present in *Shigella* and STEC. The two strains in the study sharing OgN-RKI1 (OgN-RKI1:H49 strain 16-01174 and OgN-RKI1:H20 strain 16-04698) did not show a close phylogenetic relationship, which was also indicated by their different MLST STs and H types (Fig. 4; Table 1). Homologues of OgN-RKI2, OgN-RKI3, and OgN-RKI4 were found only in E. coli (Table S3). Interestingly, five of the OgN-RKI3 homologues were serotyped as O59, but the O59 OAGC published by Guo at al. shared only 64% nucleotide identity with the new OgN-RKI3 (24) (Table S3). In addition, the *wzx* and *wzy* genes of both OAGCs were different, displaying a nucleotide identity of 72% for *wzx* and 38% for *wzy*. It appeared, however, that the OgN-RKI3 strain which harbored *stx*_2a_ showed the same MLST ST as O86:H51 strain 16-05299. One of the OgN-RKI2 homologues was found in E. coli strain P7a, serotyped as O20 by DebRoy et al. in 2016 (28). However, the O20 OAGC of strain P7a was already described by Iguchi and colleagues in 2015 (14), and the nucleotide identity between the two O20 OAGCs was only 39.5%. The *wzx* and *wzy* genes of O20 strains used by CGE SerotypeFinder also correspond to those of the O20 OAGC described by Iguchi et al. (14) and are distinct from those of the OgN-RKI2 strain (14). In two of the OgN-RKI2 homologues, the serovar was identified to be OXY24 (31) (Table S3). One of the OgN-RKI4 homologues was found in an E. coli strain with an O2-like OAGC (32). The three O2:H6 strains of MLST ST141 of this study did not share the same MLST ST and appeared on different branches of the phylogenetic tree (Fig. 4). The finding of four novel OAGCs in our study corroborates the importance of genome analysis for strain typing. Therefore, the description of further OAGCs is expected in the future, and this is of great interest to harmonize their designation. To evaluate how to handle new serotypes found by WGS studies, an international working group comprising persons with leading expertise has existed since 2017 and is hosted by Penn State University (https://sites.psu.edu/ecolishigella/).

The OAG is one of the most variable bacterial cell components. Driven by strong immunogenic selection, the types of sugars, their arrangement within the O unit, and the linkages between O units vary (33, 34). In E. coli, the OAG biosynthesis genes are clustered in the chromosome and are flanked by the colonic acid gene cluster (*wca* genes) and the histidine biosynthesis cluster (*his* genes). The genes for O unit translocation and chain synthesis, specifically, *wzx* (encoding O antigen flippase), *wzy* (encoding O antigen polymerase), and *wzm* and *wzt* (encoding components of the ABC transporter), are highly variable in sequence and are therefore especially suitable for serogroup discrimination (14, 35, 36).

Our data and those of others highlight that the OAGC distribution does not necessarily follow the phylogeny, as several serogroups are found at distinct branches of the neighbor-joining tree (Fig. 4). This supports the notion that OAGCs have been spread across E. coli by means of horizontal gene transfer and that frequent exchange may occur (14). This suggestion is also illustrated by the completely distinct genomic organization of the four novel OAGCs which we identified in this study. Only the framing of the gene cluster remains identical, but other components, such as *wzy* and *wzx*, were found at different locations with different neighboring genes (Fig. 2).

Fourteen strains were associated with severe disease, specifically, HUS and/or death, and five of those belonged to less frequently found STEC serovars, namely, O55:H7, O80:H2, O174:H21, and O177:H25 (two strains). O55:H7 strains are closely related to O157:H7 strains, and both belong to MLST ST11 (37, 38). The O55:H7 strain 17-03136 described here, which also belonged to ST11, is indeed phylogenetically close to O157:H7 strains and harbors *stx*_2a_ and *eaeA* (Fig. 4; Table 2). Those strains are considered emerging pathogens, and HUS cases associated with this serovar have been frequently described (6, 9, 39–41). Similar to the *stx*_2a_- and *eaeA*-positive O80:H2 strain 16-03025 analyzed in this study, STEC/EHEC strains of this serovar were reported in HUS patients. Due to their multidrug resistance, these strains are considered a new therapeutic challenge (9, 42, 43). The O80:H2 strains analyzed in our study also showed resistance to several antibiotics (Table S1). Zhang et al. (44) analyzed the phylogeny and phenotypes of clinical and environmental STEC O174 isolates, which may harbor distinct *fliC* H types, such as *fliC* H types H5, H21, and H46. They found that only serovar O174:H21 associates with HUS (44), and we also found this serovar in a HUS patient. The strain described here was *stx*_2d_ positive and *eaeA* negative. Cundon et al. (45) reported that O174 STEC is an emerging pathogen in Argentina. There, such strains belong to the most prevalent STEC serogroups (45). We described two O177:H25 strains (both *stx*_2c_ and *eaeA* positive) from HUS patients; one of those was classically serotyped as O177:Hnt, and the other was classically serotyped as O177:H25. An O177:Hnm and O177:Hnt strain (*stx*_2_ and *eaeA* positive) was previously isolated from an HUS patient (46).

To conclude, our data show that the typing of a large variety of STEC strains required to be typed for public health reasons can be well managed by means of genome sequence analyses. The novel WGS-based methods, moreover, enabled further analysis of strains causing severe clinical symptoms and the description of novel STEC O antigen loci, highlighting the potential of the method for detailed future investigations of common but also less frequently detected strain types.

## Supplementary Material

Supplemental file 1
