# Supplementary material for: Whole-Genome-Based Public Health Surveillance of Less Common Shiga Toxin-Producing Escherichia coli Serovars and Untypeable Strains Identifies Four Novel O Genotypes
Source: J Clin Microbiol. 2019 Sep 24;57(10):e00768-19. doi: 10.1128/JCM.00768-19 (PMC6760944; doi:10.1128/JCM.00768-19)
Supplement: Supplemental file 1 [file JCM.00768-19-s0001.pdf]

Table S1: Overview of strains used in the study (Lang et al., Whole genome-based public health surveillance of less common STEC serovars and untypable strains identifies four novel O genotypes).

Abbreviations of phenotypic resistances: ampicillin (AMP), ciprofloxacin (CIP), chloramphenicol (CMP), cefoxitin (COX), cefotiam (CTM), cefotaxime (CTX), gentamicin (GEN), kanamycin (KAN), mezlocillin (MEZ), mezlocillin/sulbactam (MSU), nalidixic acid (NAL), oxytetracycline (OTE), streptomycin (STR), sulfmethoxazole/trimethoprime (SXT)

The sequences were uploaded to the European Nucleotide Archive under the study Acc. No. PRJEB32361.

RKI No.	Phenotypic O group	Phenotypic H type	WGS derived O group	WGS derived H type	MLST	Accession No.	PCR-based stx1	WGS-derived stx1	WGS-derived stx1 subtype	PCR-based stx2	WGS-derived stx2	WGS-derived stx2 subtype	PCR-based eaeA	WGS-derived eaeA	Phenotypic resistance profile
15-02245	117	7	117	7	5292	ERS3396047 (SAMEA5591832)	+	+	stx1a	-	-	-	-	-	AMP; MEZ; OTE; SXT; STR; NAL;
15-02364	178	7	153/178	7	278	ERS3396048 (SAMEA5591833)	+	+	stx1c	+	+	stx2b	-	-	susceptible
15-02506	79	14	79	14	1249	ERS3396049 (SAMEA5591834)	-	-	-	+	+	stx2b	-	-	susceptible
15-02583	183	18	183	18	657	ERS3396050 (SAMEA5591835)	+	+	stx1a	-	-	-	-	-	susceptible
15-02585	136	16	136	16	329	ERS3396051 (SAMEA5591836)	+	+	stx1a	-	-	-	-	-	susceptible
15-02590	nt	2	80	2	301	ERS3396052 (SAMEA5591837)	-	-	-	+	+	stx2a	+	+	AMP; CMP; KAN; MEZ; OTE; SXT; STR; NAL;
15-02594	154	31	154	31	1892	ERS3396053 (SAMEA5591838)	+	+	stx1d	-	-	-	-	-	susceptible
15-02596	55	12	55	12	101	ERS3396054 (SAMEA5591839)	+	+	stx1a	-	-	-	-	-	OTE;
15-02618	45	2	45	2	301	ERS3396055 (SAMEA5591840)	-	-	-	+	+	stx2a	+	+	AMP; CMP; KAN; MEZ; OTE; STR; NAL;
15-03173	55	12	55	12	101	ERS3396056 (SAMEA5591841)	+	+	stx1a	-	-	-	-	-	AMP; CMP; MEZ; SXT; STR;
15-03191	nt	2	80	2	301	ERS3396057 (SAMEA5591842)	-	-	-	+	+	stx2a	+	+	AMP; CMP; CTM; CTX; GEN; KAN; MEZ; MSU; SXT; STR; NAL;
15-03416	nt	2	80	2	301	ERS3396058 (SAMEA5591843)	-	-	-	+	+	stx2a	+	+	AMP; CMP; GEN; KAN; MEZ; OTE; SXT; STR; NAL;
15-03726	55	12	55	12	101	ERS3396059 (SAMEA5591844)	+	+	stx1a	-	-	-	-	-	AMP; CTM; CTX; GEN; MEZ; MSU; STR;
15-03820	nt	15	23	15	70	ERS3396060 (SAMEA5591845)	-	-	-	+	+	stx2a	-	-	AMP; OTE;
15-03886	nt	30	27	30	753	ERS3396061 (SAMEA5591846)	-	-	-	+	+	stx2b	-	-	susceptible
15-04038	8	nm	8	19	201	ERS3396062 (SAMEA5591847)	-	-	-	+	+	stx2e	-	-	AMP; CTM; CTX; MEZ; OTE; SXT; STR;
15-04130	nt	30	27	30	753	ERS3396063 (SAMEA5591848)	-	-	-	+	+	stx2b	-	-	susceptible
15-04152	26	11	26	11	29	ERS3396064 (SAMEA5591849)	-	-	-	+	+	stx2a	+	+	AMP; CTM; CTX; GEN; MEZ; MSU;
15-04442	nt	8	OgN1	8	26	ERS3396065 (SAMEA5591850)	-	-	-	+	+	stx2b	-	-	susceptible
15-04722	nt	nm	89	9	10	ERS3396066 (SAMEA5591851)	-	-	-	+	+	stx2e	-	-	susceptible
15-04750	8	28	8	28	162	ERS3396067 (SAMEA5591852)	-	-	-	+	+	stx2e	-	-	OTE; STR;
15-04756	17	18	17	18	69	ERS3396068 (SAMEA5591853)	-	-	-	+	+	stx2a	-	-	susceptible
15-04760	nt	nt	89	9	10	ERS3396069 (SAMEA5591854)	-	-	-	+	+	stx2e	-	-	susceptible
15-04762	110	31	110	31	812	ERS3396070 (SAMEA5591855)	-	-	-	+	+	stx2b	-	-	susceptible
15-04827-1	nt	30	27	30	753	ERS3396071 (SAMEA5591856)	-	-	-	+	+	stx2b	-	-	susceptible
16-00119	76	19	76	19	675	ERS3396072 (SAMEA5591857)	+	+	stx1c	+	+	stx2b	-	-	susceptible
16-00120	113	4	113	4	10	ERS3396073 (SAMEA5591858)	+	+	stx1c	+	+	stx2b	-	-	susceptible
16-00135	113	4	113	4	10	ERS3396074 (SAMEA5591859)	+	+	stx1c	+	+	stx2b	-	-	susceptible
16-00137	103	2	103	2	17	ERS3396075 (SAMEA5591860)	+	+	stx1a	-	-	-	+	+	SXT;
16-00141	113	4	113	4	10	ERS3396076 (SAMEA5591861)	+	+	stx1c	+	+	stx2b	-	-	susceptible
16-00143	76	19	76	19	675	ERS3396077 (SAMEA5591862)	+	+	stx1c	+	+	stx2b	-	-	susceptible
16-00144	128	2	128	2	25	ERS3396078 (SAMEA5591863)	+	+	stx1c	+	+	stx2b	-	-	susceptible
16-00259	145	nm	145	28	32	ERS3396079 (SAMEA5591864)	-	-	-	+	+	stx2a	+	+	AMP; CTM; CTX; GEN; MEZ; STR;
16-00328	nt	2	43	2	937	ERS3396080 (SAMEA5591865)	+	+	stx1c	-	-	-	-	-	CMP; NAL;
16-00550	183	18	183	18	657	ERS3396081 (SAMEA5591866)	+	+	stx1a	+	+	stx2a	-	-	susceptible
16-00581	rauh	2	128	2	25	ERS3396082 (SAMEA5591867)	-	-	-	+	+	stx2b	-	-	susceptible
16-00596	104	7	104	7	1817	ERS3396083 (SAMEA5591868)	+	+	stx1c	-	-	-	-	-	AMP; CMP;
16-00635	178	7	153/178	7	278	ERS3396084 (SAMEA5591869)	+	+	stx1c	+	+	stx2b	-	-	susceptible
16-00636	130	11	130	11	297	ERS3396085 (SAMEA5591870)	+	+	stx1a	+	+	stx2a	-	-	AMP; CMP; CTM; CTX; MEZ; MSU;

16-00704	nt	nm	9	4	46	ERS3396086 (SAMEA5591871)	-	-	-	+	+	stx2e	-	-	susceptible
16-00707	nt	19	8	19	360	ERS3396087 (SAMEA5591872)	-	-	-	+	+	stx2e	-	-	CMP;
16-00712	23	31	23	31	101	ERS3396088 (SAMEA5591873)	+	+	stx1a	-	-	-	-	-	AMP; KAN; MEZ; OTE; STR;
16-00714	76	19	76	19	675	ERS3396089 (SAMEA5591874)	+	+	stx1c	-	-	-	-	-	CMP;
16-00717	82	nm	82	4	10	ERS3396090 (SAMEA5591875)	-	-	-	+	+	stx2d	-	-	susceptible
16-00718	128	2	128	2	25	ERS3396091 (SAMEA5591876)	-	-	-	+	+	stx2b	-	-	AMP; CMP; MEZ; OTE;
16-00910	182	nm	182	25	300	ERS3396092 (SAMEA5591877)	+	+	stx1a	-	-	-	+	+	CMP;
16-00980	63	6	63	6	583	ERS3396093 (SAMEA5591878)	-	-	-	+	+	stx2f	+	+	susceptible
16-01162	nt	21	OX18	21	40	ERS3396094 (SAMEA5591879)	+	+	stx1c	-	-	-	-	-	susceptible
16-01173	183	18	183	18	657	ERS3396095 (SAMEA5591880)	+	+	stx1a	-	-	-	-	-	susceptible
16-01174	nt	49	OgN-RKI1	49	?	ERS3396096 (SAMEA5591881)	-	-	-	+	+	stx2b	-	-	susceptible
16-01175	nt	30	27	30	753	ERS3396097 (SAMEA5591882)	-	-	-	+	+	stx2b	-	-	susceptible
16-01215	8	19	8	19	88	ERS3396098 (SAMEA5591883)	-	-	-	+	+	stx2e	-	-	CMP;
16-01258	nt	32	OgN12	32	10	ERS3396099 (SAMEA5591884)	+	+	stx1a	-	-	-	-	-	NAL;
16-01365	8	19	8	19	201	ERS3396100 (SAMEA5591885)	-	-	-	+	+	stx2e	-	-	susceptible
16-01492	181	nm	181	16	6274	ERS3396101 (SAMEA5591886)	+	+	stx1c	+	+	stx2b	-	-	susceptible
16-01499	181	49	181	49	173	ERS3396102 (SAMEA5591887)	+	+	stx1a	+	+	stx2c	-	-	susceptible
16-01506	91	19	91	14	33	ERS3396103 (SAMEA5591888)	+	+	stx1a	-	-	-	-	-	susceptible
16-01573	nt	8	OgN1	8	26	ERS3396104 (SAMEA5591889)	-	-	-	+	+	stx2b	-	-	susceptible
16-01717	57	8	OgN1	8	26	ERS3396105 (SAMEA5591890)	-	-	-	+	+	stx2b	-	-	susceptible
16-01791	43	2	43	2	937	ERS3396106 (SAMEA5591891)	+	+	stx1c	-	-	-	-	-	susceptible
16-01865	57	6	2	6	141	ERS3396107 (SAMEA5591892)	-	-	-	+	+	stx2b	-	-	susceptible
16-01921	nt	nm	100	30	993	ERS3396108 (SAMEA5591893)	-	-	-	+	+	stx2e	-	-	AMP; MEZ; OTE; SXT; STR;
16-02258	nt	nm	OgN-RKI2	16	336	ERS3396109 (SAMEA5591894)	+	+	stx1c	-	-	-	-	-	susceptible
16-02291	nt	nm	100	20	2514	ERS3396110 (SAMEA5591895)	-	-	-	+	+	stx2e	-	-	susceptible
16-02352	127	nt	127	40	10	ERS3396111 (SAMEA5591896)	+	+	stx1a	-	-	-	+	+	AMP; CMP; CTM; CTX; GEN; KAN; MEZ; MSU; OTE; SXT; STR; NAL;
16-02355	84	nm	84	2	306	ERS3396112 (SAMEA5591897)	+	+	stx1a	-	-	-	+	+	susceptible
16-02409	157	nm	157	7	11	ERS3396113 (SAMEA5591898)	-	-	-	+	+	stx2a	+	+	susceptible
16-02727	nt	nm	100	20	2514	ERS3396114 (SAMEA5591899)	-	-	-	+	+	stx2e	-		susceptible
16-02921	nt	nt	8	9	767	ERS3396115 (SAMEA5591900)	-	-	-	+	+	stx2e	-		AMP; CMP; MEZ; OTE; SXT;
16-02972	132	34	132	34	582	ERS3396116 (SAMEA5591901)	-	-	-	+	+	stx2f	+	+	susceptible
16-02991	110	31	110	31	812	ERS3396117 (SAMEA5591902)	-	-	-	+	+	stx2b	-		susceptible
16-03025	80	2	80	2	301	ERS3396118 (SAMEA5591903)	-	-	-	+	+	stx2a	+	+	AMP; CMP; KAN; MEZ; MSU; OTE; SXT; STR; NAL;
16-03031	nt	nm	100	20	2514	ERS3396119 (SAMEA5591904)	-	-	-	+	+	stx2e	-		susceptible
16-03103	rauh	7	153/178	7	278	ERS3396120 (SAMEA5591905)	+	+	stx1c	+	+	stx2b	-		AMP;
16-03160	132	34	132	34	582	ERS3396121 (SAMEA5591906)	-	-	-	+	+	stx2f	+	+	susceptible
16-03326	80	2	80	2	301	ERS3396122 (SAMEA5591907)	-	-	-	+	+	stx2a	+	+	AMP; CMP; GEN; KAN; OTE; SXT; STR; NAL;
16-03329	nt	45	12	45	?	ERS3396123 (SAMEA5591908)	+	+	stx1d	-	-	-	-		AMP;
16-03344	80	2	80	2	301	ERS3396124 (SAMEA5591909)	-	-	-	+	+	stx2a	+	+	AMP; CMP; CTM; CTX; GEN; KAN; MEZ; MSU; OTE; SXT; STR; NAL;
16-03345	8	9	8	9	23	ERS3396125 (SAMEA5591910)	-	-	-	+	+	stx2e	-		AMP; CMP;
16-03404	145	nm	145	28	32	ERS3396126 (SAMEA5591911)	-	-	-	+	+	stx2a	+	+	susceptible
16-03481	43	2	43	2	937	ERS3396127 (SAMEA5591912)	+	+	stx1c	-	-	-	-	-	AMP; NAL;
16-03485	nt	nm	8	19	88	ERS3396128 (SAMEA5591913)	-	-	-	+	+	stx2e	-	-	AMP; MEZ; SXT; STR;
16-03565	26	11	26	11	21	ERS3396129 (SAMEA5591914)	+	+	stx1a	+	+	stx2a	+	+	susceptible
16-03669	nt	nm	27	30	753	ERS3396130 (SAMEA5591915)	-	-	-	+	+	stx2b	-		susceptible
16-03680	8	28	8	28	4496	ERS3396131 (SAMEA5591916)	-	-	-	+	+	stx2a	-		susceptible
16-03686	55	7	55	9	301	ERS3396132 (SAMEA5591917)	-	-	-	+	+	stx2d	+	+	AMP; CMP; GEN; KAN; MEZ; OTE; SXT; STR; NAL;
16-03690	80	2	80	2	301	ERS3396133 (SAMEA5591918)	-	-	-	+	+	stx2a	+	+	CMP; GEN; KAN; OTE; SXT; STR; NAL;
16-03709	157	7	157	7	11	ERS3396134 (SAMEA5591919)	-	-	-	+	+	stx2a	+	+	susceptible
16-03758	145	nm	145	28	32	ERS3396135 (SAMEA5591920)	-	-	-	+	+	stx2a	+	+	susceptible
16-03919	157	7	157	7	11	ERS3396136 (SAMEA5591921)	-	-	-	+	+	stx2a	+	+	susceptible

16-03986	182	25	182	25	300	ERS3396137 (SAMEA5591922)	+	+	stx1a	-	-	-	+	+	susceptible
16-04050	125	6	125	6	583	ERS3396138 (SAMEA5591923)	-	-	-	+	+	stx2f	+	+	susceptible
16-04102	43	2	43	2	937	ERS3396139 (SAMEA5591924)	+	+	stx1c	+	+	stx2b	-	-	NAL;
16-04112	106	18	106	18	663	ERS3396140 (SAMEA5591925)	-	-	-	+	+	stx2d	-	-	susceptible
16-04141	nt	6	2	6	141	ERS3396141 (SAMEA5591926)	-	-	-	+	+	stx2b	-	-	susceptible
16-04147	rauh	nm	153/178	7	4975	ERS3396142 (SAMEA5591927)	+	+	stx1c	+	+	stx2b	-	-	susceptible
16-04148	169	21	81	21	737	ERS3396143 (SAMEA5591928)	+	+	stx1c	+	+	stx2b	-	-	susceptible
16-04178	54	21	OgN-RKI3	21	155	ERS3396144 (SAMEA5591929)	-	-	-	+	+	stx2a	-	-	OTE; STR;
16-04225	38	26	38	26	10	ERS3396145 (SAMEA5591930)	+	+	stx1c	+	+	stx2b	-	-	OTE; STR;
16-04226	38	26	38	26	10	ERS3396146 (SAMEA5591931)	+	+	stx1c	+	+	stx2b	-	-	OTE; STR;
16-04233	166	28	166	28	1819	ERS3396147 (SAMEA5591932)	-	-	-	+	+	stx2b	-	-	susceptible
16-04280	2	6	2	6	141	ERS3396148 (SAMEA5591933)	-	-	-	+	+	stx2b	-	-	susceptible
16-04388	109	21	109	21	40	ERS3396149 (SAMEA5591934)	-	-	-	+	+	stx2f	+	+	susceptible
16-04414	rauh	nm	166	28	1819	ERS3396150 (SAMEA5591935)	+	+	stx1c	-	-	-	-	-	susceptible
16-04417	183	18	183	18	657	ERS3396151 (SAMEA5591936)	+	+	stx1a	+	+	stx2d	-	-	susceptible
16-04503	80	2	80	2	301	ERS3396152 (SAMEA5591937)	-	-	-	+	+	stx2d	+	+	AMP; CMP; KAN; MEZ; OTE; SXT; STR; NAL;
16-04698	nt	2	112	2	388	ERS3396153 (SAMEA5591938)	+	+	stx1c	-	-	-	-	-	susceptible
16-04798	nt	40	168	8, 40	718	ERS3396154 (SAMEA5591939)	-	-	-	+	+	stx2a	-	-	OTE; STR;
16-04840	nt	40	OgN1	8, 40	26	ERS3396155 (SAMEA5591940)	-	-	-	+	+	stx2b	-	-	susceptible
16-04846	nt	nt	OgN-RKI1	20	6060	ERS3396156 (SAMEA5591941)	+	+	stx1c	-	-	-	-	-	susceptible
16-04847	80	2	80	2	301	ERS3396157 (SAMEA5591942)	-	-	-	+	+	stx2a	+	+	AMP; CMP; KAN; MEZ; OTE; SXT; STR; NAL;
16-04865	nt	nt	27	30	753	ERS3396158 (SAMEA5591943)	-	-	-	+	+	stx2b	-	-	susceptible
16-04944	rauh	nm	3	12	329	ERS3396159 (SAMEA5591944)	+	+	stx1a	-	-	-	-	-	susceptible
16-04982	165	nm	165	25	119	ERS3396160 (SAMEA5591945)	+	+	stx1a	+	+	stx2a	+	+	susceptible
16-05089	rauh	2	43	2	937	ERS3396161 (SAMEA5591946)	+	+	stx1c	-	-	-	-	-	susceptible
16-05108	nt	31	110	31	812	ERS3396162 (SAMEA5591947)	-	-	-	+	+	stx2b	-	-	susceptible
16-05138	nt	nm	183	18	657	ERS3396163 (SAMEA5591948)	+	+	stx1a	+	+	stx2a	-	-	susceptible
16-05184	nt	20	OgN10	20	691	ERS3396164 (SAMEA5591949)	+	+	stx1a	-	-	-	-	-	susceptible
16-05299	nt	nm	86	51	155	ERS3396165 (SAMEA5591950)	+	+	stx1a	-	-	-	-	-	AMP; CIP; CMP; CTM; CTX; MEZ; MSU; OTE; STR; NAL;
16-05329	nt	2	112	2	388	ERS3396166 (SAMEA5591951)	+	+	stx1c	-	-	-	-	-	AMP;
16-05332	rauh	16	181	16	6274	ERS3396167 (SAMEA5591952)	+	+	stx1c	-	-	-	-	-	susceptible
16-05416	nt	nm	100	20	2514	ERS3396168 (SAMEA5591953)	-	-	-	+	+	stx2e	-	-	AMP; MEZ; OTE; STR;
17-00140-1	157	nm	157	7	11	ERS3396169 (SAMEA5591954)	+	+	stx1a	-	-	-	+	+	susceptible
17-00242	36	19	36	19	10	ERS3396170 (SAMEA5591955)	-	-	-	+	+	stx2e	-	-	susceptible
17-00261	157	7	157	7	11	ERS3396171 (SAMEA5591956)	+	+	stx1a	+	+	stx2a	+	+	susceptible
17-00285	26	11	26	11	29	ERS3396172 (SAMEA5591957)	-	-	-	+	+	stx2a	+	+	AMP; MEZ; STR;
17-00402	36	14	36	14	1176	ERS3396173 (SAMEA5591958)	-	-	-	+	+	stx2e	-	-	susceptible
17-00411	36	nm	36	14	1176	ERS3396174 (SAMEA5591959)	-	-	-	+	+	stx2g	-	-	susceptible
17-00416	rauh	nm	181	16	6274	ERS3396175 (SAMEA5591960)	+	+	stx1c	+	+	stx2b	-	-	susceptible
17-00475	156	25	156	25	300	ERS3396176 (SAMEA5591961)	-	-	-	+	+	stx2a	+	+	susceptible
17-00641	nt	25	177	25	659	ERS3396177 (SAMEA5591962)	-	-	-	+	+	stx2c	+	+	OTE; STR;
17-00703	81	21	81	21	737	ERS3396178 (SAMEA5591963)	+	+	stx1c	-	-	-	-	-	AMP; MEZ; STR;
17-00772	157	nm	157	7	11	ERS3396179 (SAMEA5591964)	-	-	-	+	+	stx2a	+	+	susceptible
17-00778	91	14	91	14	33	ERS3396180 (SAMEA5591965)	+	+	stx1a	+	+	stx2b	-	-	CMP;
17-00844	186	10	123/186	10	3695	ERS3396181 (SAMEA5591966)	+	+	stx1c	+	+	stx2b	-	-	susceptible
17-00847	104	21	104	21	672	ERS3396182 (SAMEA5591967)	+	+	stx1a	+	+	stx2d	-	-	susceptible
17-00856	157	nm	157	7	11	ERS3396183 (SAMEA5591968)	+	+	stx1a	+	+	stx2a	+	+	susceptible
17-00882	157	nm	157	7	11	ERS3396184 (SAMEA5591969)	+	+	stx1a	+	+	stx2c	+	+	susceptible
17-00884	157	7	157	7	11	ERS3396185 (SAMEA5591970)	-	-	-	+	+	stx2a	+	+	susceptible
17-00946	103	2	103	2	17	ERS3396186 (SAMEA5591971)	+	+	stx1a	-	-	-	+	+	susceptible
17-00991	103	2	103	2	17	ERS3396187 (SAMEA5591972)	+	+	stx1a	-	-	-	+	+	susceptible

17-00995	36	14	36	14	1176	ERS3396188 (SAMEA5591973)	-	-	-	+	+	stx2g	-	-	susceptible
17-00997	36	19	36	19	10	ERS3396189 (SAMEA5591974)	-	-	-	+	+	stx2g	-	-	susceptible
17-00998	36	14	36	14	1176	ERS3396190 (SAMEA5591975)	-	-	-	+	+	stx2g	-	-	susceptible
17-01000	36	14	36	14	1176	ERS3396191 (SAMEA5591976)	-	-	-	+	+	stx2g	-	-	CMP;
17-01045	157	nm	157	7	11	ERS3396192 (SAMEA5591977)	+	+	stx1a	+	+	stx2c	+	+	AMP; MEZ; STR;
17-01061	26	11	26	11	21	ERS3396193 (SAMEA5591978)	-	-	-	+	+	stx2a	+	+	AMP; MEZ;
17-01142	157	nm	157	7	11	ERS3396194 (SAMEA5591979)	+	+	stx1a	+	+	stx2c	+	+	STR;
17-01185	177	nm	177	25	659	ERS3396195 (SAMEA5591980)	-	-	-	+	+	stx2c	+	+	susceptible
17-01359	157	7	157	7	11	ERS3396196 (SAMEA5591981)	-	-	-	+	+	stx2c	+	+	susceptible
17-01749	103	2	103	2	17	ERS3396197 (SAMEA5591982)	+	+	stx1a	-	-	-	+	+	susceptible
17-01774	104	nm	104	21	672	ERS3396198 (SAMEA5591983)	+	+	stx1a	+	+	stx2a	-	-	susceptible
17-01864	157	7	157	7	11	ERS3396199 (SAMEA5591984)	-	-	-	+	+	stx2a	+	+	susceptible
17-01972	157	nm	157	7	587	ERS3396200 (SAMEA5591985)	-	-	-	+	+	stx2a	+	+	susceptible
17-01975	145	28	145	28	32	ERS3396201 (SAMEA5591986)	-	-	-	+	+	stx2a	+	+	susceptible
17-02088	157	nm	157	7	1804	ERS3396202 (SAMEA5591987)	-	-	-	+	+	stx2c	+	+	susceptible
17-02233	157	7	157	7	11	ERS3396203 (SAMEA5591988)	+	+	stx1a	+	+	stx2a	+	+	CMP;
17-02566	157	7	157	7	11	ERS3396204 (SAMEA5591989)	-	-	-	+	+	stx2c	+	+	susceptible
17-02598	157	nm	157	7	11	ERS3396205 (SAMEA5591990)	-	-	-	+	+	stx2a	+	+	susceptible
17-02628	157	7	157	7	11	ERS3396206 (SAMEA5591991)	-	-	-	+	+	stx2a	+	+	susceptible
17-02676	157	7	157	7	11	ERS3396207 (SAMEA5591992)	+	+	stx1a	+	+	stx2c	+	+	AMP; CMP; GEN; MEZ; OTE; SXT; STR; NAL;
17-02726	157	7	157	7	11	ERS3396208 (SAMEA5591993)	+	+	stx1a	+	+	stx2c	+	+	susceptible
17-02778	157	7	157	7	11	ERS3396209 (SAMEA5591994)	-	-	-	+	+	stx2a	+	+	susceptible
17-02862	157	7	157	7	11	ERS3396210 (SAMEA5591995)	-	-	-	+	+	stx2c	+	+	susceptible
17-02938	157	7	157	7	11	ERS3396211 (SAMEA5591996)	+	+	stx1a	+	+	stx2c	+	+	susceptible
17-03030	nt	21	174	21	677	ERS3396212 (SAMEA5591997)	-	-	-	+	+	stx2d	-	-	susceptible
17-03136	55	7	55	7	335	ERS3396213 (SAMEA5591998)	-	-	-	+	+	stx2a	+	+	SXT; STR;
17-03548	103	2	103	2	17	ERS3396214 (SAMEA5591999)	+	+	stx1a	-	-	-	+	+	susceptible
17-03780	157	7	157	7	11	ERS3396215 (SAMEA5592000)	-	-	-	+	+	stx2c	+	+	susceptible
17-03836	157	7	157	7	11	ERS3396216 (SAMEA5592001)	+	+	stx1a	+	+	stx2c	+	+	susceptible
17-03846	157	7	157	7	11	ERS3396217 (SAMEA5592002)	+	+	stx1a	+	+	stx2a	+	+	susceptible
17-03961	157	7	157	7	11	ERS3396218 (SAMEA5592003)	-	-	-	+	+	stx2c	+	+	susceptible
17-03963	157	7	157	7	11	ERS3396219 (SAMEA5592004)	+	+	stx1a	+	+	stx2a	+	+	susceptible
17-03964	157	7	157	7	11	ERS3396220 (SAMEA5592005)	-	-	-	+	+	stx2c	+	+	susceptible
17-04023	157	7	157	7	11	ERS3396221 (SAMEA5592006)	-	-	-	+	+	stx2a	+	+	susceptible
17-04111	157	7	157	7	11	ERS3396222 (SAMEA5592007)	+	+	stx1a	-	+	stx2c	+	+	susceptible
17-04143	157	7	157	7	11	ERS3396223 (SAMEA5592008)	+	+	stx1a	+	+	stx2c	+	+	susceptible
17-04868	157	7	157	7	11	ERS3396224 (SAMEA5592009)	+	+	stx1a	+	+	stx2c	+	+	susceptible
17-04959	157	7	157	7	11	ERS3396225 (SAMEA5592010)	+	+	stx1a	+	+	stx2a	+	+	susceptible
17-05089	157	7	157	7	11	ERS3396226 (SAMEA5592011)	-	-	-	+	+	stx2a	+	+	susceptible
17-05090	103	11	103	11	723	ERS3396227 (SAMEA5592012)	+	+	stx1a	-	-	-	+	+	AMP; CTM; CTX; MEZ; MSU; OTE; SXT; STR;
17-05135	nt	2	OX18	2	4017	ERS3396228 (SAMEA5592013)	+	+	stx1a	+	+	stx2a	-	-	susceptible
17-05203	rauh	16	87	16	2101	ERS3396229 (SAMEA5592014)	-	-	-	+	+	stx2b	-	-	susceptible
17-05261	nt	nm	141	19	8237	ERS3396230 (SAMEA5592015)	-	-	-	+	+	stx2e	-	-	susceptible
17-05292	nt	36	7	31	446	ERS3396231 (SAMEA5592016)	+	+	stx1c	-	-	-	-	-	susceptible
17-05333	181	16	181	16	6274	ERS3396232 (SAMEA5592017)	+	+	stx1c	+	+	stx2b	-	-	susceptible
17-05371	43	2	43	2	937	ERS3396233 (SAMEA5592018)	+	+	stx1c	-	-	-	-	-	susceptible
17-05374	157	7	157	7	11	ERS3396234 (SAMEA5592019)	-	-	-	+	+	stx2a	+	+	susceptible
17-05375	nt	49	OgN31	49	2520	ERS3396235 (SAMEA5592020)	-	-	-	+	+	stx2a	-	-	susceptible
17-05381	113	21	113	21	223	ERS3396236 (SAMEA5592021)	-	-	-	+	+	stx2a	-	-	susceptible
17-05419	157	7	157	7	11	ERS3396237 (SAMEA5592022)	+	+	stx1a	+	+	stx2c	+	+	susceptible
17-05433	157	7	157	7	11	ERS3396238 (SAMEA5592023)	+	+	stx1a	+	+	stx2c	+	+	susceptible

17-05493	nt	30	27	30	753	ERS3396239 (SAMEA5592024)	-	-	-	+	+	stx2b	-	-	susceptible
17-05504	nt	52	187	52	642	ERS3396240 (SAMEA5592025)	+	+	stx1c	-	-	-	-	-	AMP; COX;
17-05507	109	25	182	25	300	ERS3396241 (SAMEA5592026)	+	+	stx1a	-	-	-	+	+	susceptible
17-05609	nt	nm	100	30	993	ERS3396242 (SAMEA5592027)	-	-	-	+	+	stx2e	-		OTE;
17-05662	63	6	63	6	583	ERS3396243 (SAMEA5592028)	-	-	-	+	+	stx2f	+	+	susceptible
17-05663	nt	30	27	30	753	ERS3396244 (SAMEA5592029)	-	-	-	+	+	stx2b	-		susceptible
17-05667	nt	2	80	2	301	ERS3396245 (SAMEA5592030)	-	-	-	+	+	stx2a	+	+	AMP; CMP; GEN; KAN; MEZ; OTE; SXT; STR; NAL;
17-05676	nt	29	OgN-RKI4	29	515	ERS3396246 (SAMEA5592031)	-	-	-	+	+	stx2b	-		susceptible
17-05752	157	7	157	7	11	ERS3396247 (SAMEA5592032)	-	-	-	+	+	stx2c	+	+	susceptible
17-05760	98	nm	98	21	306	ERS3396248 (SAMEA5592033)	+	+	stx1a	-	-	-	+	+	susceptible
17-05763	157	7	157	7	11	ERS3396249 (SAMEA5592034)	-	-	-	+	+	stx2a	+	+	susceptible
17-05810	nt	21	OX18	21	40	ERS3396250 (SAMEA5592035)	+	+	stx1c	-	-	-	-	-	CMP; NAL;
17-05813	110	31	110	31	812	ERS3396251 (SAMEA5592036)	-	-	-	+	+	stx2b	-	-	susceptible
17-05826	nt	7	117	7	504	ERS3396252 (SAMEA5592037)	+	+	stx1a	-	-	-	-	-	AMP;
17-05857	63	6	63	6	583	ERS3396253 (SAMEA5592038)	-	-	-	+	+	stx2f	+	+	susceptible
17-05859	rauh	7	153/178	7	278	ERS3396254 (SAMEA5592039)	+	+	stx1c	+	+	stx2b	-	-	susceptible
17-05861	nt	30	100	30	993	ERS3396255 (SAMEA5592040)	-	-	-	+	+	stx2e	-	-	OTE;
17-05863	nt	18	15	18	69	ERS3396256 (SAMEA5592041)	+	+	stx1a	-	-	-	-	-	STR;
17-05864	rauh	7	153/178	7	4975	ERS3396257 (SAMEA5592042)	+	+	stx1c	+	+	stx2b	-	-	susceptible
17-05865	nt	8	179	8	297	ERS3396258 (SAMEA5592043)	-	-	-	+	+	stx2a	-	-	susceptible
17-05936	nt	16	OgN-RKI2	16	336	ERS3396259 (SAMEA5592044)	+	+	stx1c	-	-	-	-	-	susceptible
17-06065	76	19	76	19	675	ERS3396260 (SAMEA5592045)	+	+	stx1c	-	+	stx2b	-	-	CMP;
17-06072	nt	4	78	4	3101	ERS3396261 (SAMEA5592046)	+	+	stx1c	-	-	-	-	-	susceptible
17-06075	nt	8	174	8	13	ERS3396262 (SAMEA5592047)	+	+	stx1c	+	+	stx2b	-	-	susceptible
17-06108	nt	16	OgN13	16	295	ERS3396263 (SAMEA5592048)	+	+	stx1c	-	-	-	-	-	susceptible
17-06200	nt	4	78	4	3101	ERS3396264 (SAMEA5592049)	+	+	stx1c	-	-	-	-	-	susceptible
17-06201	nt	30	27	30	753	ERS3396265 (SAMEA5592050)	-	-	-	+	+	stx2b	-	-	susceptible
17-06322	nt	21	174	21	677	ERS3396266 (SAMEA5592051)	-	-	-	+	+	stx2d	-	-	susceptible
17-06327	63	6	63	6	583	ERS3396267 (SAMEA5592052)	-	-	-	+	+	stx2f	+	+	susceptible
17-06334	156	25	156	25	4942	ERS3396268 (SAMEA5592053)	+	+	stx1a	-	-	-	+	+	susceptible
17-06335	111	8	111	8	16	ERS3396269 (SAMEA5592054)	+	+	stx1a	+	+	stx2a	+	+	CMP; OTE;
17-06351	nt	48	OgN31	48	3519	ERS3396270 (SAMEA5592055)	-	-	-	+	+	stx2a	-	-	susceptible
17-06369	136	nm	136	20	398	ERS3396271 (SAMEA5592056)	+	+	stx1c	-	-	-	-	-	susceptible
17-06460	7	4	7	4	93	ERS3396272 (SAMEA5592057)	+	-	-	-	-	-	-	-	susceptible
17-06523	43	nt	43	2	937	ERS3396273 (SAMEA5592058)	+	+	stx1c	-	-	-	-	-	susceptible
17-06526	nt	7	117	7	504	ERS3396274 (SAMEA5592059)	+	+	stx1a	-	-	-	-	-	AMP; MEZ; OTE; SXT; STR;
17-07220	8	14	8	14	136	ERS3396275 (SAMEA5592060)	-	-	-	+	+	stx2b	-	-	susceptible
17-07221	8	14	8	14	136	ERS3396276 (SAMEA5592061)	-	-	-	+	+	stx2b	-	-	susceptible
17-07255	103	2	103	2	17	ERS3396277 (SAMEA5592062)	+	+	stx1a	-	-	-	+	+	susceptible
17-07259	8	4	8	4	?	ERS3396278 (SAMEA5592063)	-	-	-	+	+	stx2e	-	-	susceptible

Table S2: Reference sequences for O-AGCs downloaded from NCBI. (Lang et al., Whole genome-based public health surveillance of less common STEC serovars and untypable strains identifies four novel O genotypes)

O-AGC/ virulence marker gene	GenBank No./ Accession No.
OgN1	LC125927.1
OgN8	LC125928.1
OgN9	LC125929.1
OgN10	LC125930.1
OgN12	LC125931.1
OgN31	LC125932.1
OgN2	LC177546.1
OgN3	LC177547.1
OgN4	LC177548.1
OgN5	LC177549.1
OgN13	LC177550.1
OgN14	LC177551.1
OgN15	LC177552.1
OgN16	LC177553.1
OgN17	LC177554.1
OX18	KJ710507.1
OX21	KJ739596.2
OX38	KJ739599.3
OX10	KJ778792.1
OX6	KJ778794.1
OX9	KJ778795.1
OX13	KP710591.1
OX25	KP710594.1
OX19	KP868751.1
OX28	KT207929.1

Tab. S3: Homologues of the new O-AGCs found by NCBI nBLAST (Lang et al., Whole genome-based public health surveillance of less common STEC serovars and untypable strains identifies four novel O genotypes). n.d.=not defined.

**Homologues to new O-AGC OgN-RKI1**				
strain	Serotype	isolation source	nucleotide identity [%]	GenBank Accession
S. boydii BS984	19	human feces	98.27	PUGO00000000.1
S. boydii 83-578	19	n.d.	98.28	CP026814.1
STEC2841	Ont:H20	human feces	97.93	LOIQ01000034.1
**Homologuesto new O-AGC OgN-RKI2**				
strain	Serotype	isolation source	nucleotide identity [%]	GenBank Accession
E. coli MOD1-3827	OXY24:H16	cheese	100	NZ_NJRY01000017.1
E. coli MOD1-3831	OXY24:H17	cheese	100	NZ_NJRW01000017.1
E. coli UMEA 3176-1	n.d.	n.d.	99.99	AWCA01000017.1
E. coli MOD1-6444	n.d.	deer	99.96	NZ_NOQO01000009.1

E. coli MOD1-6465	n.d.	pig serum	99.97	NZ_NNVR01000001.1
E. coli 116 2DZ3	n.d.	dog	99.96	MOJG01000001.1
E. coli 495 BN4	n.d.	fly	99.96	NZ_MOHI01000022.1
E. coli B132	n.d.	human feces	99.98	NZ_NRYW01000024.1
E. coli P7a	O20:H-	n.d.	99.98	KJ778793.1
E. coli MOD1-5481	n.d.	water	98.32	NZ_NLOH01000004.1
**Homologues to new O-AGC OgN-RKI3**				
strain	Serotype	isolation source	nucleotide identity [%]	GenBank Accession
E. coli 31-678	n.d.	human urine	99.98	LENM01000002.1
E. coli Lys36 c175	n.d.	soil	99.98	PJRE01000168.1
E. coli Lys45 c120	n.d.	soil	99.98	PJRD01000116.1
E. coli Lys53 c131	n.d.	soil	99.98	PJRB01000125.1
E. coli Lys24 c153	n.d.	soil	99.98	PJRI01000151.1
E. coli Lys28 c153	n.d.	soil	99.98	PJRH01000152.1
E. coli Lys34 c165	n.d.	soil	99.98	PJRG01000163.1
E. coli Lys35 c166	n.d.	soil	99.98	PJRF01000150.1
E. coli Lys52 c132	n.d.	soil	99.98	PJRC01000131.1
E. coli C019	O59:H21	human	99.98	NWBD01000002.1

		feces		
E. coli C322	O59:H21	human feces	99.98	NWAU01000002.1
E. coli C323	O59:H21	human feces	99.98	NWAT01000002.1
E. coli F426	Ont:Hnt	human feces	99.98	NWAK01000017.1
E. coli T168	O59:H21	human feces	99.98	NWAB01000006.1
E. coli T297	O59:H21	human feces	99.98	NVZZ01000007.1
**Homologues new O-AGC OgN-RKI4**				
strain	Serotype	isolation source	nucleotide identity [%]	GenBank Accession
E. coli N38833PS	n.d.	farm	99.99	JUBT01000041.1
E. coli YH17162	n.d.	chicken	99.99	PTMY01000002.1
E. coli MOD1-5029	n.d.	beef	99.97	NLUJ01000004.1
E. coli A185	n.d.	human feces	99.88	NRZN01000085.1
E. coli AVC113	Ont:H21	chicken	99.87	QOGQ01000002.1
E. coli M856	Ont:H6	human feces	99.15	FBUQ01000014.1

E. coli ERS085421SC	n.d.	human feces	98.6	CYDG01000036.1
E. coli ERS085427SC	n.d.	human feces	98.61	CYDD01000037.1
E. coli 303139 26	n.d.	human feces	98.51	JHTB01000026.1
E. coli O2-like O-AGC	O2:H49	n.d.	77.14	KY115228.1
